# A Comparison of Symptoms in Hospitalized African American and White Persons With Dementia

**DOI:** 10.1093/geroni/igaa057.2754

**Published:** 2020-12-16

**Authors:** Elizabeth Galik, Boltz Marie, Rachel Arendacs, Ashley Kuzmik

**Affiliations:** 1 University of Maryland School of Nursing, Ellicott City, Maryland, United States; 2 Penn State, University Park, Pennsylvania, United States; 3 Penn State University College of Nursing, State College, Pennsylvania, United States; 4 Pennsylvania State University, University Park, Pennsylvania, United States

## Abstract

There exist significant race disparities in the prevalence of dementia, with black persons with dementia (PWD) showing higher co-morbidity and more frequent hospitalizations, yet little is known how clinical presentations compare. This study compared physical function, delirium, depressive symptoms, and behavioral and psychological symptoms of distress (BPSD) in black and white PWDs when hospitalized. A multivariate analysis of covariance showed that, controlling for age, gender, cognitive status, and comorbidities, black PWD had more delirium (mean= 3.8, SD= 2.9) as compared to white PWDs (mean=2.4, SD= 2.2, F=4.8, p =.029). Additionally, black PWD had more depressive symptoms (mean= 11.7, SD= 6.7) as compared to white PWD (mean = 9.0, SD= 5.2, F=6.6, p =.011), and less improvement in functional status admission to discharge (mean =12.4, SD= 18.9) as compared to white PWD (mean=17.8, SD=18.8, F=12.3, p=.001). There were no differences in BPSD. Continued research examining factors influencing differences in race cohorts is warranted.

